# Shifting Trends in Intensive Cardiovascular Care Unit Admission Patterns: Retrospective Insights and Prospective Implications

**DOI:** 10.3390/diagnostics15202563

**Published:** 2025-10-11

**Authors:** Ranel Loutati, Louay Taha, Mohammad Karmi, Noam Fink, Pierre Sabouret, Mamas A. Mamas, Ari Naimark, Ariella Tvito, Yonit Wiener-Well, Amjad Abu-Salman, Mony Shuvy, Ofer Merin, Michael Glikson, Elad Asher

**Affiliations:** 1 Jesselson Integrated Heart Center, The Eisenberg R&D Authority, Shaare Zedek Medical Center, and Faculty of Medicine, Hebrew University of Jerusalem, Jerusalem 9103102, Israel; ranellout@gmail.com (R.L.); louayt@szmc.org.il (L.T.); mkarmi@szmc.org.il (M.K.); ariellat@szmc.org.il (A.T.); yonitw@szmc.org.il (Y.W.-W.); amjadabu@szmc.org.il (A.A.-S.); monysh@szmc.org.il (M.S.); mglikson@szmc.org.il (M.G.); 2 Assuta Medical Centers, Faculty of Medicine, Tel Aviv University, Tel Aviv 6329302, Israel; noamfink@bezeqint.net; 3 ACTION Study Group, Institut de Cardiologie, Hôpital Pitié-Salpêtrière, Sorbonne Université, 75005 Paris, France; cardiology.sabouret@gmail.com; 4 National College of French Cardiologists, 13 Rue Niepce, 75014 Paris, France; 5 Keele Cardiovascular Research Group, Centre for Prognosis Research, Keele University, Stoke-on-Trent ST5 5BG, UK; mamasmamas1@yahoo.co.uk; 6 Department of Cardiothoracic Surgery, Shaare Zedek Medical Center and Faculty of Medicine, Hebrew University of Jerusalem, Jerusalem 9103102, Israel; arina@szmc.org.il; 7 Shaare Zedek Medical Center, Hebrew University of Jerusalem, Jerusalem 9103102, Israel; merin@szmc.org.il

**Keywords:** intensive cardiovascular care unit, admission trends, one-year mortality

## Abstract

**Background**: Intensive Cardiovascular Care Units (ICCUs) are critical in managing high-acuity cardiovascular conditions, yet contemporary data on evolving admission patterns and their association with outcomes are limited. **Methods**: We conducted a retrospective cohort study of all patients admitted to a tertiary-care ICCU between July 2019 and December 2024. Patients were stratified by admission period: early (2019–2021) and late (2022–2024). Baseline characteristics, index diagnosis, interventions, complications, and mortality outcomes were compared. The primary endpoints were in-hospital and one-year mortality. **Results**: The study included 6266 patients (median age 69 years, 32% female). Of them, 3125 and 3141 patients were admitted in the early and late periods, respectively. Patients in the later period exhibited a higher burden of co-morbidities, including increased rates of atrial fibrillation, cognitive impairment, and dialysis (*p* < 0.05 for all). The pattern of index diagnoses shifted, showing an increase in heart failure (5.6% vs. 3.7%, *p* = 0.001) and malignant arrhythmia admissions (13.9% vs. 9.3%, *p* < 0.001), alongside a decline in cases of NSTEMI and pulmonary embolism. The use of urgent percutaneous coronary intervention, transcatheter valvular interventions, and microaxial pumps increased, whereas intra-aortic balloon pump usage declined. In-hospital mortality remained consistent between the periods at 2.7%. However, adjusted one-year mortality was significantly reduced in the later period (adjusted HR 0.84, 95% CI 0.71–0.98, *p* = 0.037). **Conclusions**: Over five years, ICCU admissions showed increasing complexity and evolving procedural trends. Despite higher acuity, adjusted one-year survival improved, highlighting care advances and the value of continuous data-driven ICCU optimization.

## 1. Introduction

Cardiovascular disease (CVD) remains the leading global cause of death and disability, accounting for over 18 million deaths annually and imposing a substantial burden on healthcare systems worldwide [1]. Recent projections suggest a continued rise in both the incidence and prevalence of CVD, largely driven by aging populations, increasing rates of cardiometabolic comorbidities, and persistent disparities in cardiovascular care delivery across regions and populations [1,2,3,4]. In this evolving landscape, the role of the intensive cardiovascular care units (ICCU) is expected to become even more critical. Traditionally designed to manage acute coronary syndromes (ACS), the ICCU now accommodates a broader spectrum of cardiovascular emergencies, including advanced heart failure, malignant arrhythmias, high-risk procedural recovery, and cardiogenic shock [5,6]. The demand for high-intensity cardiovascular care is projected to escalate further, in conjunction with the expected population aging and rising CVD incidence [7], underscoring the importance of understanding how ICCU utilization and case complexity are evolving in response to shifting epidemiologic trends.

Despite the increasing role of the ICCU as a central hub for high-acuity cardiovascular care, contemporary data describing evolving admission patterns and their relationship with clinically meaningful outcomes remain limited. While a few studies have explored temporal changes in patient characteristics, procedural trends, and care delivery models [8,9], the link between these shifts and patient outcomes, particularly mortality, has not been thoroughly established.

Hence, we analyzed a large, contemporary tertiary care center ICCU cohort to evaluate trends in admissions patterns, procedural profiles, complications, and outcomes of patients with complex cardiovascular diseases to guide strategic planning and improve workforce readiness for the upcoming era of intensive cardiovascular care.

## 2. Methods

### 2.1. Study Population

This study was a retrospective single-center observational cohort study that was performed in a tertiary care center ICCU. The study population consisted of non-selected consecutive patients admitted to the ICCU between 1 July 2019 and 31 December 2024. All patients admitted during the study period were included. If a patient was admitted multiple times, the first hospitalization was selected.

### 2.2. Clinical Data and Study Outcome

Data were anonymously documented in the ICCU by the local coordinator and prospectively submitted to an electronic case report form (eCRF). Data was checked for accuracy and out-of-range values by the coordinating unit, and any case of inconsistency was addressed. Demographic data, presenting symptoms, comorbid conditions, and physical examination were systematically recorded. Laboratory, imaging, angiographic results, and clinical course data were collected as well [10]. Patients were divided into two identical time groups based on the period of admission, with 1 April 2022 being the date cutoff: early period: 1 July 2019–31 March 2022 (33 months), late period: 1 April 2022–31 December 2024 (33 months). The main diagnosis for each patient was determined by the treating physician according to the European society of Cardiology (ESC) guidelines for major myocardial injury causes [11,12]. If a patient presented with multiple diagnoses, only one was considered as the primary diagnosis, prioritized in the following order: ST-segment elevation myocardial infarction (STEMI), non-STEMI (NSTEMI), exacerbation of congestive heart failure (CHF), pulmonary embolism (PE), peri/myocarditis, arrhythmia (tachyarrhythmia or bradyarrhythmia), or post-cardiac procedures. If a patient did not fall into any of these categories, their primary diagnosis was designated as “other.” The primary outcomes of the current study were in-hospital and one-year all-cause mortality. Survival data were available for all included subjects from the Israeli Population Register up to 1 January 2025. There were no missing data. The Institutional Review Board (IRB) approved the study based on strict maintenance of participants’ anonymity by de-identifying during database analysis (approval number 0233-19-SZMC). Consent was waived by the Institutional IRB due to the retrospective and observational nature of the study. The authors have no conflicts of interest to declare, and no funding was applied to the study.

### 2.3. Statistical Analysis

Continuous variables were expressed as mean  ±  standard deviation if normally distributed or median with interquartile range if skewed. Categorical variables were presented as frequency (%). Differences in demographics, baseline characteristics, main diagnosis, treatment, and complications between the two groups of patients were studied. Comparison of means was performed using Student’s *t*-test or Mann-Whitney U-test where appropriate. Statistical comparison of the differences in categorical data between the two groups was performed using the chi-square test or Fisher’s exact test. For survival analysis patients were censored only in the case of death during follow-up. The probability of death at one year according to the study groups was graphically displayed according to the method of Kaplan–Meier, with a comparison of cumulative survival across strata by the log-rank test. Univariable and multivariable Cox proportional hazards regression models were used to compare patients between the two periods. The multivariable model was constructed using stepwise forward selection based on the likelihood ratio test, incorporating variables that were significant in the univariable analysis or are known to influence mortality in ICCU patients. The adjusted cox model incorporated the following variables: age, sex, CHF, atrial fibrillation (AF), cognitive decline, chronic kidney disease (CKD) defined as eGFR < 30 mL/min/1.73m^2^, previous MI, and ejection fraction (EF) from the admission echocardiogram. The proportional hazards assumption was assessed graphically and tested using Schoenfeld residuals (Supplementary Table S1). For the one-year survival analysis, patients admitted after 1 January 2024 were excluded to ensure a uniform follow-up duration. A sensitivity analysis was conducted after excluding patients with confirmed COVID-19 infection. All analyses were performed using R software version 4.3.3 (R Foundation for Statistical Computing).

## 3. Results

### 3.1. Number of Admissions and Baseline Characteristics

**Figure 1:**
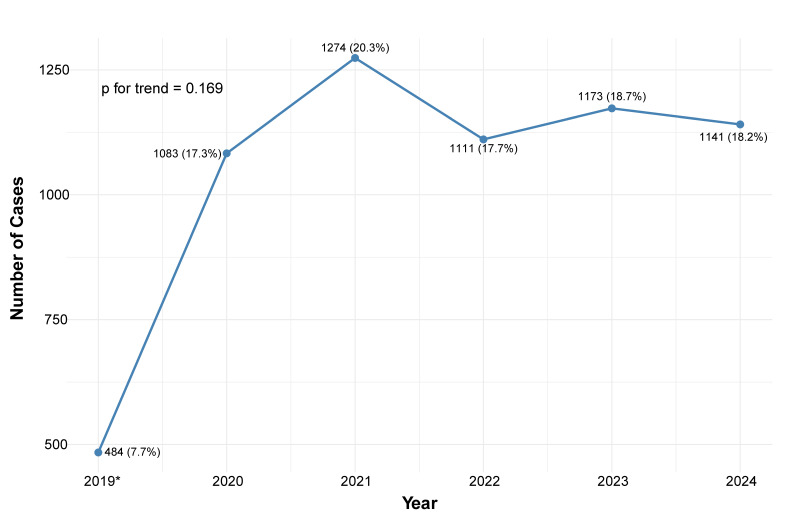
Number of intensive cardiovascular care unit admissions per year. Plot of number of admissions per year since the start of registry. * Registry started in July 2019.

The study population included 6,266 patients, with a median age of 69 years [interquartile range (IQR): 58–79; min-max: 15-102], of whom 1990 (32%) were female. The number of admissions per year was similar across periods, with 3125 patients in the early period and 3141 in the late period. Annual admission trends are illustrated in Figure 1. Table 1 presents the baseline characteristics of patients in both periods. Overall, baseline characteristics were comparable between groups, with no significant differences in age, sex, and length of stay. However, patients in the late period had more family history of coronary artery disease (CAD) (6.7% vs. 8.2%, *p* = 0.024), and higher prevalence of atrial fibrillation (15.1% vs. 16.9%, *p* = 0.047), cardiac implantable electronic devices (CIED) (5.9% vs. 7.6%, *p* = 0.008), cognitive decline (3.3% vs. 4.3%, *p* = 0.037, and the need for dialysis treatment (2.0% vs. 2.8%, *p* = 0.033).

### 3.2. Main Diagnoses on Admission

**Figure 2:**
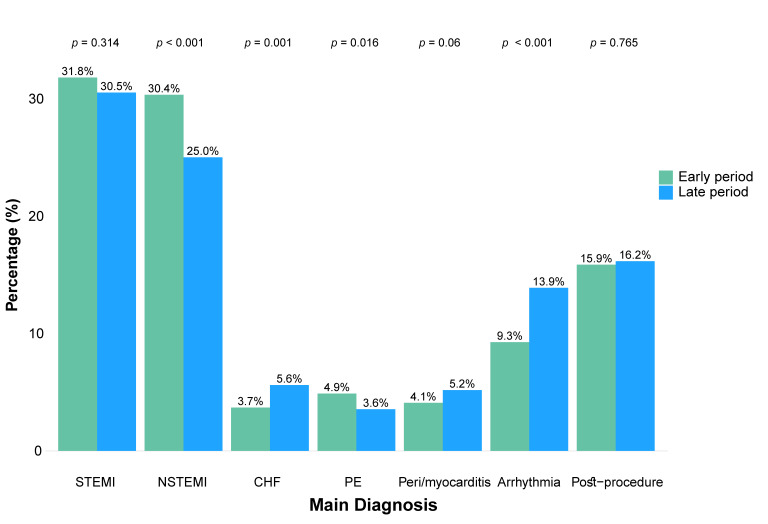
Bar plot of cases’ main diagnoses divided into early and late periods. This bar plot demonstrates the relative portion of the main diagnoses in the ICCU in each of the periods, showing that in the late period more patients were hospitalized in the ICCU due to CHF, peri/myocarditis and life-threatening arrhythmias. CHF = Congestive Heart Failure; NSTEMI = Non-ST-Elevation Myocardial Infarction; PE = Pulmonary Embolism; STEMI = ST segment Elevation Myocardial Infarction.

The distribution of main admission diagnoses is shown in Figure 2. Compared to patients admitted during the early period, those in the late period had higher rates of admissions for CHF (3.7% vs. 5.6%, *p* = 0.001) and tachyarrhythmias or bradyarrhythmias (9.3% vs. 13.9%, *p* < 0.001), similar rates of STEMI (31.8% vs. 30.5%, *p* = 0.314) and post-procedural monitoring (15.9% vs. 16.2%, *p* = 0.765), and significantly lower rates of NSTEMI (30.4% vs. 25.0%, *p* < 0.001) and PE (4.9% vs. 3.6%, *p* = 0.016).

### 3.3. Interventions and Complications

Interventions and in-hospital complications are summarized in Table 2 and Table 3, respectively.

Patients in the late period underwent urgent percutaneous coronary interventions (PCIs) more frequently as compared with patients from the early period (27.1% vs. 24.5%, *p* = 0.035), as well as more transcatheter valvular interventions including transcatheter aortic valve implantation (TAVI) (9.4% vs. 7.2%, *p* = 0.002) and mitral transcatheter edge-to-edge repair (TEER) (2.4% vs. 1%, *p* < 0.001). Interestingly, late period patients received more blood transfusions due to anemia or significant bleeding (5.9% vs. 2.2%, *p* < 0.001).

Notably, the use of intra-aortic balloon pumps (IABP) declined significantly (2.4% vs. 0.9%, *p* < 0.001), while the use of microaxial flow pumps (Impella) increased (0.3% vs. 0.8%, *p* = 0.005). In terms of complications, there was an increase in the incidence of left ventricular (LV) thrombus (1.1% vs. 0.5%, *p* = 0.016) and vascular complications (2.3% vs. 1.4%, *p* = 0.021). However, there were fewer cases of cardiogenic shock developing during ICCU stay (9.2% vs. 6.5%, *p* = 0.014) and fewer cases of acute kidney injury (AKI) (4.6% vs. 3.1%, *p* = 0.003).

### 3.4. In-Hospital and One-Year Mortality Rates

During ICCU stay, a total of 173 patients (2.7%) died during hospitalization, with similar rates observed in the early and late periods [86 (2.7%) vs. 87 (2.7%), respectively]. Cox regression analysis showed that the period of admission was not associated with in-hospital mortality in either univariable analysis (HR 0.98, 95% CI 0.73–1.33, *p* > 0.9) or multivariable analysis (adjusted HR 1.06, 95% CI 0.75–1.51, *p* = 0.7). The full results are summarized in Table 4a.

**Figure 3:**
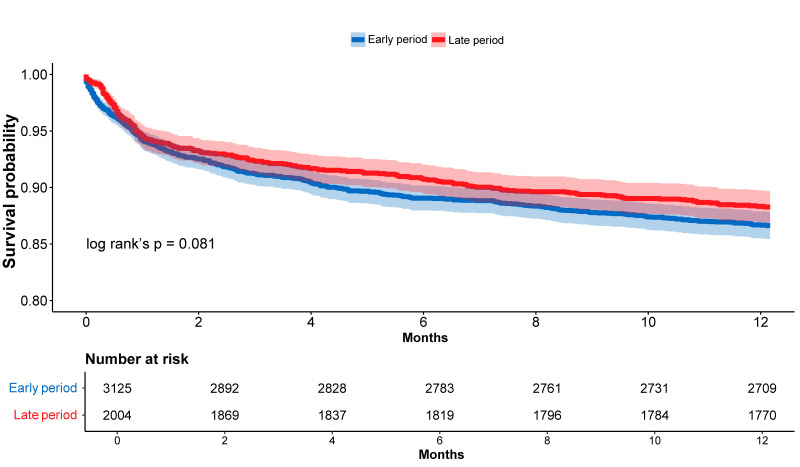
Kaplan–Meier Curves for One-Year Survival. Kaplan–Meier curves for one-year survival demonstrating that although in-hospital survival rates were similar between periods (2.7% for both), for one-year survival there was a trend towards a higher probability to survive in the late period. However, it was not statistically significant (log-rank’s *p* = 0.081).

**Figure 4:**
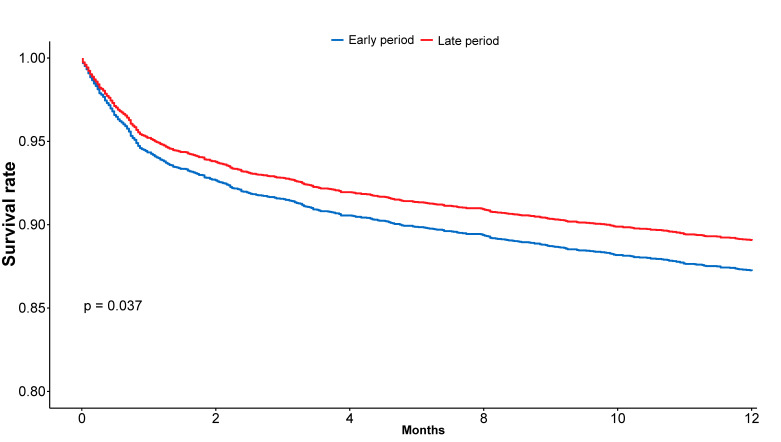
Adjusted Cox survival Curves for One-Year Survival. This figure presents adjusted one-year survival curves derived from a multivariable Cox proportional hazards model, stratified by admission period. The multivariable Cox model is adjusted for age, sex, AF, CHF, CKD, cognitive decline, previous MI, and EF, demonstrating that compared to patients in the early period, those in the late period had 16% reduction in mortality risk (HR 0.84, 95% CI 0.71–0.98, *p* = 0.037). AF = Atrial Fibrillation; CHF = Congestive Heart Failure; CKD = Chronic Kidney Disease; EF = Ejection Fraction; MI = Myocardial Infarction.

One-year survival analysis was conducted after excluding patients admitted after 1 January 2024, resulting in a cohort of 5129 patients: 3125 in the early period and 2004 in the late period. Within this cohort, one-year mortality occurred in 418 patients (13.4%) in the early period and 235 patients (10.5%) in the late period. Kaplan–Meier analysis demonstrated higher one-year survival rates in the late period group compared with the early period group, although it was borderline significance (86.6% ± 0.61% vs. 88.3% ± 0.72%, respectively; log-rank *p* = 0.081; Figure 3). Although univariable Cox analysis did not show a statistically significant association between admission period and mortality (HR 0.87, 95% CI 0.74–1.02, *p* = 0.081), multivariable analysis revealed that admission during the late period was independently associated with a 16% reduction in one-year mortality risk (adjusted HR 0.84, 95% CI 0.71–0.98, *p* = 0.037) as shown in Figure 4. Additional predictors of one-year mortality in the multivariable analysis are presented in Table 4b.

To ensure the generalizability of our findings, a sensitivity analysis excluding patients with confirmed COVID-19 infection during ICCU admission was conducted. A total of 106 patients (3.4%) in the early period and 12 patients (0.4%) in the late period tested positive for COVID-19. After excluding these individuals, multivariable analysis yielded consistent results, demonstrating a 15% reduction in one-year mortality among patients admitted during the late period compared to those in the early period (adjusted HR 0.85, 95% CI 0.70–0.99, *p* = 0.041).

## 4. Discussion

This retrospective cohort study evaluated evolving trends in intensive cardiovascular care by comparing patients admitted to a tertiary-care ICCU between two distinct periods: early (July 2019–March 2022) and late (April 2022–December 2024). The analysis of 6266 patients provides valuable insights into how case complexity, procedural practices, and outcomes have shifted over time. Our findings reveal an evolution in ICCU admissions, with increasingly complex patient profiles, broader use of invasive and advanced treatments, and similar short-term mortality rates with improved one-year survival that was borderline by Kaplan–Meier but significant in adjusted Cox regression. These results underscore the adaptability of modern cardiovascular intensive care and highlight areas for continued refinement and resource allocation.

Despite stable admission volumes between the early and late study periods, there was a shift toward a more complex and frail patient population in the later period, reflected by higher rates of cognitive decline and dialysis at baseline. Diagnostic patterns evolved, with an increase in admissions due to CHF and arrhythmia, and a decline in NSTEMI and PE presentations. Procedural trends demonstrated greater utilization of urgent PCI, transcatheter valvular interventions, and Impella devices, alongside a marked reduction in IABP use. Importantly, in-hospital mortality remained stable over time, while adjusted one-year survival improved significantly in the late period, with multivariable analysis confirming a 16% relative reduction in mortality risk. These findings highlight a progressive shift in ICCU case complexity and management, accompanied by improved long-term outcomes despite the increasing acuity of care.

Concurrently, cardiovascular care is undergoing rapid transformation, marked by a sharp increase in the use of advanced interventional procedures and device-based therapies. Innovations in structural heart interventions, complex PCI, and mechanical circulatory support have contributed to improved patient outcomes but also introduced new challenges in post-procedural care [8,13,14]. Patients undergoing transcatheter valve interventions, revascularization for complex or high-risk coronary disease, and advanced heart failure therapies, often with the use of temporary mechanical circulatory support, frequently require intensive hemodynamic monitoring and multidisciplinary care, making ICCU admission essential in many cases [15,16]. Importantly, the ICCU also manages a substantial proportion of non-cardiac conditions, including sepsis, acute kidney injury, and acute respiratory failure, which are increasingly prevalent among its patients [17,18]. These evolving clinical demands have significantly reshaped the acuity and case mix of ICCU admissions, underscoring the need for contemporary reassessment of patient profiles, resources, and care models.

Numerous studies have examined trends in ICCU patient profiles over the past decade, consistently reporting an increasing prevalence of older adults and individuals with complex comorbidities [19,20,21]. In a large, recent study from France, Guillermou et al. [21] analyzed ICCU admissions between 2014 and 2023 and reported a rise in heart failure cases, while rates of acute coronary syndromes remained stable. Our detailed cohort confirms and extends these findings, demonstrating not only an increased frequency of heart failure admissions but also a significant rise in arrhythmia-related admissions. Concurrently, we observed a decline in NSTEMI admissions to the ICCU, likely reflecting evolving triage practices that direct lower-risk patients to general cardiology and internal medicine wards rather than intensive care settings. Furthermore, we observed an improvement in adjusted one-year survival among patients admitted during the late period, which may plausibly reflect advancements in treatment strategies and a reduction in complication rates, as supported by our data.

Our survival analysis revealed a consistency in short-term (in-hospital) mortality across both periods, while demonstrating a favorable trend toward lower one-year mortality in recent years, with multivariable Cox regression confirming that admission during the late period was independently associated with improved survival. The stable in-hospital mortality rates (2.7%), despite rising case complexity and procedural risk, suggest that ICCUs have maintained high-quality acute care delivery even as patient acuity has increased. This stability may reflect early recognition of clinical deterioration, timely implementation of evidence-based interventions, and greater use of hemodynamic monitoring and advanced support devices, which are key factors for management of patients in the ICCU [8,9]. Importantly, most in-hospital deaths occurred within the first hours of admission, indicating that many patients arrived in critical condition, possibly due to pre-hospital delays or late presentation. These patterns may reflect the severity of illness at admission rather than reflecting ICCU performance.

To our knowledge, this is the first contemporary study to demonstrate a temporal reduction in adjusted one-year mortality among ICCU patients. Several factors may underlie the observed improvement in long-term outcomes. These include center-level advancements, such as enhanced training of clinical staff and the implementation of standardized, guideline-based ICCU protocols. Additionally, broader paradigm shifts in cardiovascular care likely contributed, including the increased use of microaxial flow pumps (Impella), which provide superior hemodynamic support and have been linked to improved outcomes [22]. Other contributing elements may include improved care strategies for complex populations such as older and frail patients [7], greater interdisciplinary coordination among cardiology subspecialties [23], and the establishment of dedicated response teams for high-risk conditions such as pulmonary embolism [24] and cardiogenic shock [25]. These advances collectively represent a shift toward more protocol-driven, multidisciplinary care that may be responsible for improving one-year outcomes in this high-risk population. This may also reflect better post-discharge care, with implementation of guideline-directed medical therapy for conditions such as heart failure, myocardial infarction, and arrhythmias using beta-blockers, angiotensin receptor-neprilysin inhibitors (ARNIs), sodium-glucose cotransporter-2 (SGLT2) inhibitors, and implantable devices when appropriate. The increasing adoption of these interventions may have contributed to greater clinical stability following discharge [26]. In addition, the development and expansion of transitional care programs after acute hospitalizations have also been associated with reductions in both readmissions [27] and mortality [28].

Given the rising burden of comorbidities observed among patients in the later period of our study, there may be a benefit in incorporating validated prognostic tools into routine clinical assessment. Scores such as the Charlson Comorbidity Index [29] and the CHA_2_DS_2_-VASc score [30], originally developed to predict long-term mortality and thromboembolic risk, respectively, may offer valuable insight beyond their traditional contexts. Their integration into the management of hospitalized patients, particularly in the ICCU, could improve risk stratification, guide clinical decision-making, and support more personalized care for complex, multimorbid patients. Using these tools may also facilitate early identification of high-risk individuals and promote resource optimization within high-acuity settings.

While the findings from this study may not have immediate clinical implications, they offer important insights into the evolving landscape of ICCU care and help anticipate future demands. As cardiovascular patients become increasingly complex, with rising rates of heart failure, device therapy, and multisystem comorbidities, ICCU units must adapt accordingly. Based on our experience and findings, this includes expanding expertise in dialysis and mechanical circulatory support (MCS), routinely involving other specialties such as nephrology and geriatrics, and ensuring the implementation of standardized, evidence-based care protocols.

### 4.1. Study Strengths and Limitations

This study has several important strengths. First, it leverages a large, real-world cohort of over 6000 unselected patients admitted to a tertiary-care ICCU, providing a comprehensive and contemporary assessment of evolving trends in case complexity, interventions, and outcomes. Second, it includes both in-hospital and one-year mortality data with complete follow-up, offering an extended view of patient trajectories. Finally, the use of multivariable Cox regression allowed adjustment for key clinical confounders, enhancing the validity of observed associations.

However, several limitations should be acknowledged. First, this was a single-center study, potentially limiting external validity due to institutional practice patterns and referral bias. Nevertheless, the ICCU studied serves over 1000 admissions annually, representing a robust high-volume center. Additionally, although the early and late cohorts were each 33 months in duration and one-year survival was consistently assessed, the greater number of patients with available follow-up in the early period may have introduced bias. Second, part of the early period overlapped with the COVID-19 pandemic, which may have influenced admission patterns and outcomes (such as fewer PE diagnoses in the late period), although sensitivity analysis excluding patients who were COVID-19 positive showed consistent results. Third, mortality outcomes were limited to all-cause death, as cause-specific mortality was not available; however, Israeli national mortality patterns closely resemble those in the European Union, where cardiovascular death remains a leading cause [31]. Fourth, our analysis lacked certain granular clinical and laboratory variables such as Killip class, NYHA functional class, and natriuretic peptides levels, which may have contributed to residual confounding. Fifth, while multivariable modeling allowed adjustment for key covariates, the observational design precludes establishing causality. Finally, the growing proportion of post-structural interventional cases in recent years, many of whom are ambulatory and admitted for short-term observation, may have partially attenuated the overall acuity profile and should be considered when interpreting mortality trends.

### 4.2. Conclusions

Over five years, ICCU patients’ complexity increased with evolving diagnoses and procedural trends. Notably, one-year mortality improved despite higher patient acuity. These findings underscore the need for ongoing evaluation of ICCU practices, infrastructure, and multidisciplinary care to meet the demands of an increasingly complex cardiovascular population. Further research is warranted to identify specific care processes and interventions most strongly associated with improved long-term survival.

## Figures and Tables

**Table 1:** Patients’ baseline characteristics.

Variable	Early Period (N = 3125)	Late Period (N = 3141)	*p*-Value
Age (years)	69.0 (58.0–79.0)	69.0 (58.0–79.0)	0.301
Female sex	960 (30.7%)	1030 (32.8%)	0.082
BMI (kg/m^2^)	27.3 (24.2–30.9)	27.2 (24.3–30.7)	0.634
Hypertension—no. (%)	1904 (60.9%)	1909 (60.8%)	0.923
Dyslipidemia—no. (%)	1632 (52.2%)	1651 (52.6%)	0.808
Diabetes—no. (%)	1132 (36.2%)	1201 (38.2%)	0.105
Smoking—no. (%)	848 (27.1%)	793 (25.2%)	0.094
Family History of CAD—no. (%)	210 (6.7%)	259 (8.2%)	0.024
Prior CAD—no. (%)	958 (30.7%)	957 (30.5%)	0.893
Prior CABG—no. (%)	198 (6.3%)	235 (7.5%)	0.082
CVA/TIA—no. (%)	230 (7.4%)	223 (7.1%)	0.727
PAD—no. (%)	145 (4.6%)	140 (4.5%)	0.774
CHF—no. (%)	516 (16.5%)	512 (16.3%)	0.848
AFIB—no. (%)	471 (15.1%)	532 (16.9%)	0.047
COPD—no. (%)	263 (8.4%)	255 (8.1%)	0.703
Pulmonary Hypertension—no. (%)	158 (5.1%)	170 (5.4%)	0.564
CIED—no. (%)	184 (5.9%)	238 (7.6%)	0.008
Cognitive Decline—no. (%)	102 (3.3%)	135 (4.3%)	0.037
Debilitated—no. (%)	105 (3.4%)	131 (4.2%)	0.105
Malignancy—no. (%)	272 (8.7%)	270 (8.6%)	0.915
Anemia—no. (%)	157 (5.0%)	185 (5.9%)	0.146
CKD—no. (%)	416 (13.3%)	408 (13.0%)	0.734
Dialysis—no. (%)	61 (2.0%)	88 (2.8%)	0.033
Length of stay (days)	2 (1–3)	2 (1–3)	0.151

Values are median (Interquartile range: [Q1–Q3]) for continuous variables, and number of occurrences (frequency %) for categorical variables. AFIB = Atrial Fibrillation; BMI = Body mass index; CABG = Coronary Artery Bypass Graft surgery; CAD = Coronary Artery Disease; CHF = Congestive Heart Failure; CIED = Cardiac Implantable Electronic Device; CKD = Chronic Kidney Disease; COPD = Chronic Obstructive Pulmonary Disease; CVA = Cerebro-Vascular Accident; PAD = Peripheral Artery Disease; TIA = Transient Ischemic Attack.

**Table 2:** Treatments and interventions during intensive cardiovascular care unit Admission.

Intervention	Early Period (N = 3125)	Late Period (N = 3141)	*p*-Value
Diagnostic Cath.—no. (%)	354 (11.3%)	410 (13.1%)	0.040
Urgent PCI	765 (24.5%)	852 (27.1%)	0.035
PCI—no. (%)	550 (17.6%)	504 (16.0%)	0.107
CABG—no. (%)	57 (1.8%)	76 (2.4%)	0.159
TAVI—no. (%)	226 (7.2%)	296 (9.4%)	0.002
Mitral TEER—no. (%)	33 (1.0%)	78 (2.5%)	<0.001
Ablation/CIED implantation—no. (%)	327 (10.5%)	335 (10.7%)	0.827
Pulmonary Thrombolysis/aspiration—no. (%)	13 (0.4%)	23 (0.7%)	0.136
Blood transfusion—no. (%)	69 (2.2%)	185 (5.9%)	<0.001
CPR—no. (%)	127 (4.1%)	109 (3.5%)	0.243
Mechanical Ventilation—no. (%)	276 (8.8%)	255 (8.1%)	0.333
IABP—no. (%)	75 (2.4%)	29 (0.9%)	<0.001
Impella—no. (%)	8 (0.3%)	25 (0.8%)	0.005
ECMO—no. (%)	12 (0.4%)	15 (0.5%)	0.710
TTM—no. (%)	33 (1.1%)	35 (1.1%)	0.920

Values are number of occurrences (frequency %) for categorical variables. CIED = Cardiac Implantable Electronic Device; CPR = Cardio-Pulmonary Resuscitation; ECMO = Extra-Corporeal Membrane Oxygenation; IABP = Intra-Aortic Balloon Pump; PCI = Percutaneous Coronary Intervention; TAVI = Transcatheter Aortic Valve Implantation; TEER = Transcatheter Edge-to-Edge Repair; TTM = Targeted Temperature Management.

**Table 3:** Complications during intensive cardiovascular care unit admission.

Complication	Early Period (N = 3125)	Late Period (N = 3141)	*p*-Value
Malignant arrhythmia—no. (%)	77 (2.5%)	69 (2.2%)	0.537
Cardiogenic shock *—no. (%)	287 (9.2%)	203 (6.5%)	0.014
Mechanical Complication (VSR/rupture)—no. (%)	17 (0.5%)	15 (0.5%)	0.848
LV Thrombus—no. (%)	16 (0.5%)	34 (1.1%)	0.016
Septic shock *—no. (%)	58 (1.9%)	70 (2.2%)	0.341
Stroke—no. (%)	36 (1.2%)	26 (0.8%)	0.242
AKI—no. (%)	143 (4.6%)	98 (3.1%)	0.003
Significant bleeding—no. (%)	124 (4.0%)	116 (3.7%)	0.616
Vascular complication—no. (%)	45 (1.4%)	71 (2.3%)	0.020
Anoxic brain damage—no. (%)	10 (0.3%)	21 (0.7%)	0.074

Values are number of occurrences (frequency %) for categorical variables. AKI = Acute Kidney Injury; LV = Left Ventricle; VSR = Ventricular Septal Rupture. * not presented initially.

**Table 4:** (**a**): Univariable and multivariable Cox regression analyses for in-hospital mortality. (**b**): Univariable and Multivariable Cox regression Analyses for One-Year Mortality.

(a)			
Univariable			
Characteristic	HR	95% CI	*p*-value
Late period	0.98	0.73–1.33	>0.9
**Multivariable**			
**Characteristic**	**HR**	**95% CI**	***p*-value**
Late period	1.06	0.75–1.51	0.7
Age (per year)	1.06	1.05–1.08	<0.001
Male gender	0.53	0.36–0.76	<0.001
Prior MI	1.61	1.11–2.33	0.012
AF	1.31	0.87–1.96	0.2
CHF	1.24	0.85–1.81	0.3
CKD	1.59	0.74–3.44	0.2
Cognitive decline	1.51	0.78–2.89	0.2
EF (per %)	0.96	0.94–0.98	<0.001
**(b)**			
**Univariable**			
**Characteristic**	**HR**	**95% CI**	***p*-value**
Late period	0.90	0.80–1.02	0.11
**Multivariable**			
**Characteristic**	**HR**	**95% CI**	***p*-value**
Late period	0.84	0.71–0.98	0.037
Age (per year)	1.06	1.05–1.06	<0.001
Male gender	0.78	0.68–0.88	<0.001
Prior MI	1.26	1.12–1.43	<0.001
AF	1.42	1.17–1.71	<0.001
CHF	1.98	1.75–2.25	<0.001
CKD	3.08	2.44–3.90	<0.001
Cognitive decline	1.52	1.10–2.09	0.011
EF (per %)	0.98	0.97–0.98	<0.001

AF = Atrial Fibrillation; CHF = Congestive Heart Failure; CKD = Chronic Kidney Disease; EF = Ejection Fraction; MI = Myocardial Infarction.

## Data Availability

The data underlying this article will be shared on reasonable request to the corresponding author.

## Supplementary Materials

The following supporting information can be downloaded at: https://www.mdpi.com/article/10.3390/diagnostics15202563/s1, Table S1: Schoenfeld residuals test for the assumption of proportional hazard.

## Abbreviations

ICCU	Intensive Cardiovascular Care Unit
