# Supplementary material for: Shifting Trends in Intensive Cardiovascular Care Unit Admission Patterns: Retrospective Insights and Prospective Implications
Source: Diagnostics (Basel). 2025 Oct 11;15(20):2563. doi: 10.3390/diagnostics15202563 (PMC12564537; doi:10.3390/diagnostics15202563)
Supplement: Supplementary file 1 [file diagnostics-15-02563-s001.zip › diagnostics-3921969-supplementary.pdf]

Supplemental material for:

Shifting Trends in Intensive Cardiovascular Care Unit Admission Patterns: Retrospective Insights and Prospective Implications

Ranel Loutati [1], Louay Taha [1], Mohammad Karmi [1], Noam Fink [2], Pierre Sabouret [3,4], Mamas A. Mamas [5], Ari Naimark [6], Ariella Tvito [1], Yonit Wiener-Well [1], Amjad Abu-Salman [1], Mony Shuvy [1], Ofer Merin [7], Michael Glikson [1] and Elad Asher [1,*]

1. Jesselson Integrated Heart Center, The Eisenberg R&D Authority, Shaare Zedek Medical Center, and Faculty of Medicine, Hebrew University of Jerusalem, 9103102 Jerusalem, Israel; ranellout@gmail.com (R.L.); louayt@szmc.org.il (L.T.); mkarmi@szmc.org.il (M.K.); ariellat@szmc.org.il (A.T.); yonitw@szmc.org.il (Y.W.-W); amjadabu@szmc.org.il (A.A.-S.); monysh@szmc.org.il (M.S.); mglikson@szmc.org.il (M.G.)
2. Assuta Medical Centers, Faculty of Medicine, Tel Aviv University, 6329302 Tel Aviv, Israel; noamfink@bezeqint.net
3. ACTION Study Group, Institut de Cardiologie, Hôpital Pitié-Salpêtrière, Sorbonne Université, 75005 Paris, France; cardiology.sabouret@gmail.com
4. National College of French Cardiologists, 13 Rue Niepce, 75014 Paris, France
5. Keele Cardiovascular Research Group, Centre for Prognosis Research, Keele University, Stoke-on-Trent ST5 5BG, UK; mamasmamas1@yahoo.co.uk
6. Department of Cardiothoracic Surgery, Shaare Zedek Medical Center and Faculty of Medicine, Hebrew University of Jerusalem,9103102 Jerusalem , Israel; arina@szmc.org.il
7. Shaare Zedek Medical Center, Hebrew University of Jerusalem, Jerusalem 9103102, Israel; merin@szmc.org.il

* Correspondence: easher@szmc.org.il; Tel.: +972-52-5-046-080

Supplemental Table S1: Schoenfeld residuals test for the assumption of proportional hazard

**Covariate**	**Schoenfeld residuals p-value**	**Is the assumption of proportional hazard met?**
Period	0.982	Yes
Age (per year)	0.109	Yes
Gender	0.113	Yes
Prior MI	0.217	Yes
AF	0.300	Yes
CHF	0.162	Yes
CKD	0.130	Yes
Cognitive decline	0.815	Yes
EF (per %)	0.064	Yes

AF = Atrial Fibrillation; CHF = Congestive Heart Failure; CKD = Chronic Kidney Disease; EF = Ejection Fraction; MI = Myocardial Infarction

We evaluated the assumption of proportional hazard by testing Schoenfeld residuals. P>0.05 means that the proportional hazard assumption is met.
